# In-Depth Examination of the Functionality and Performance of the Internet Hospital Information Platform: Development and Usability Study

**DOI:** 10.2196/54018

**Published:** 2024-11-08

**Authors:** Guang-Wei Zhang, Bin Li, Zheng-Min Gu, Wei-Feng Yang, Yi-Ran Wang, Hui-Jun Li, Han-Bing Zheng, Ying-Xu Yue, Kui-Zhong Wang, Mengchun Gong, Da-Xin Gong

**Affiliations:** 1 Department of Smart Hospital The First Hospital of China Medical University Shenyang China; 2 The Internet Hospital Branch of the Chinese Research Hospital Association Beijing China; 3 Department of Information Center The First Hospital of China Medical University Shenyang China; 4 Shenyang Medical & Film Science and Technology Co Ltd Shenyang China; 5 Enduring Medicine Smart Innovation Research Institute Shenyang China; 6 ZuoYi Technology Co Ltd Beijing China; 7 YLZ Ruitu Information Technology Co Ltd Guangzhou China; 8 iMEDWAY Technology Co Ltd Beijing China; 9 Digital Health China Co Ltd Beijing China

**Keywords:** internet hospital, smart hospital, mobile applications, operational data, information system, online service, patient service, management tool, electronic prescriptions, medical education, integration

## Abstract

**Background:**

Internet hospitals (IHs) have rapidly developed as a promising strategy to address supply-demand imbalances in China’s medical industry, with their capabilities directly dependent on information platform functionality. Furthermore, a novel theory of “Trinity” smart hospital has provided advanced guidelines on IH constructions.

**Objective:**

This study aimed to explore the construction experience, construction models, and development prospects based on operational data from IHs.

**Methods:**

Based on existing information systems and internet service functionalities, our hospital has built a “Smart Hospital Internet Information Platform (SHIIP)” for IH operations, actively to expand online services, digitalize traditional health care, and explore health care services modes throughout the entire process and lifecycle. This article encompasses the platform architecture design, technological applications, patient service content and processes, health care professional support features, administrative management tools, and associated operational data.

**Results:**

Our platform has presented a set of data, including 82,279,669 visits, 420,120 online medical consultations, 124,422 electronic prescriptions, 92,285 medication deliveries, 6,965,566 prediagnosis triages, 4,995,824 offline outpatient appointments, 2025 medical education articles with a total of 15,148,310 views, and so on. These data demonstrate the significant role of IH as an indispensable component of our physical hospital services, with deep integration between online and offline health care systems.

**Conclusions:**

The upward trends in various data metrics indicate that our IH has gained significant recognition and usage among both the public and healthcare workers, and may have promising development prospects. Additionally, the platform construction approach, which prioritizes comprehensive service digitization and the 'Trinity' of the public, healthcare workers, and managers, serves as an effective means of promoting the development of Internet Hospitals. Such insights may prove invaluable in guiding the development of IH and facilitating the continued evolution of the Internet healthcare sector.

## Introduction

Internet hospitals (IHs) are an innovative health care service model pioneered in the Chinese medical industry, which have experienced rapid development in recent years, especially after the outbreak of the COVID-19 pandemic [1-6]. As of May 2023, the number of approved IHs has exceeded 3000, with an overwhelming number and recognition in the public ones [7]. They have been regarded as a promising strategy to alleviate the supply-demand imbalance and shape the future development of the medical industry, due to their potential to offer improved accessibility, efficient resource utilization, enhanced doctor-patient communication, enable personalized medicine, support public health emergencies, and facilitate medical innovation [8-10]. However, the current operational conditions of many IHs are far from ideal, even in a “zombie state” [11]. The reasons that may account for this situation, include low enthusiasm among health care professionals, limited functionalities and service offerings of IHs, as well as insufficient construction experience among managers without sufficient successful case references [2].

It has been widely acknowledged that internet-based medical platforms are pivotal elements in the construction of IHs, as they form the fundamental infrastructure for the operation of IHs and directly influence the successful implementation and development of IHs [4,8]. Since the launch of the first internet-based diagnosis and treatment platform in 2014 [1], IHs in China have undergone a decade of exploration and a clear and improved evolution in terms of their concept, functional positioning, construction models, and management regulations. Strictly speaking, IHs are no longer confined to online consultations alone, rather they are medical institutions that use the internet to provide medical and related services. In an ideal state, IHs should maximize the networked realization of physical hospital functions, even encompassing all the functions of physical hospitals online. Moreover, a theory of “Trinity” smart hospital construction has been recently developed in China, which emphasizes an intelligence and collaborative development of “smart service” for the people, “smart medicine” for medical workers, and “smart management” for hospital managers [12]. As pioneers in smart hospital initiatives, IHs will be further enhanced in the theoretical framework and functional positioning depending on this new theory [12].

Following these guiding principles, The First Hospital of China Medical University has pioneered the planning, design, and implementation of a comprehensive platform for IHs, called the “Smart Hospital Intelligent Internet Platform (SHIIP).” By leveraging existing information systems (eg, hospital information system and picture archiving and communication system) and internet service functionalities, the platform has undergone progressive development and integration, demonstrating initial effectiveness. This article comprehensively presents our construction experience of this platform, covering its conceptual framework, objectives, overall planning, technological applications, functional achievements, construction process, and application outcomes.

## Methods

### Overview

The SHIIP provides a comprehensive, humanized, patient-centered medical service system for patients, and also provides comprehensive support for medical staff members and hospital administrators. The details are elaborated in Multimedia Appendix 1, including Objective and Concept, Blueprint of the SHIIP, Technical Architecture, Network Structure, and Data Service Architecture. The functionalities will be described from the perspectives of different users as follows.

**Figure 1:**
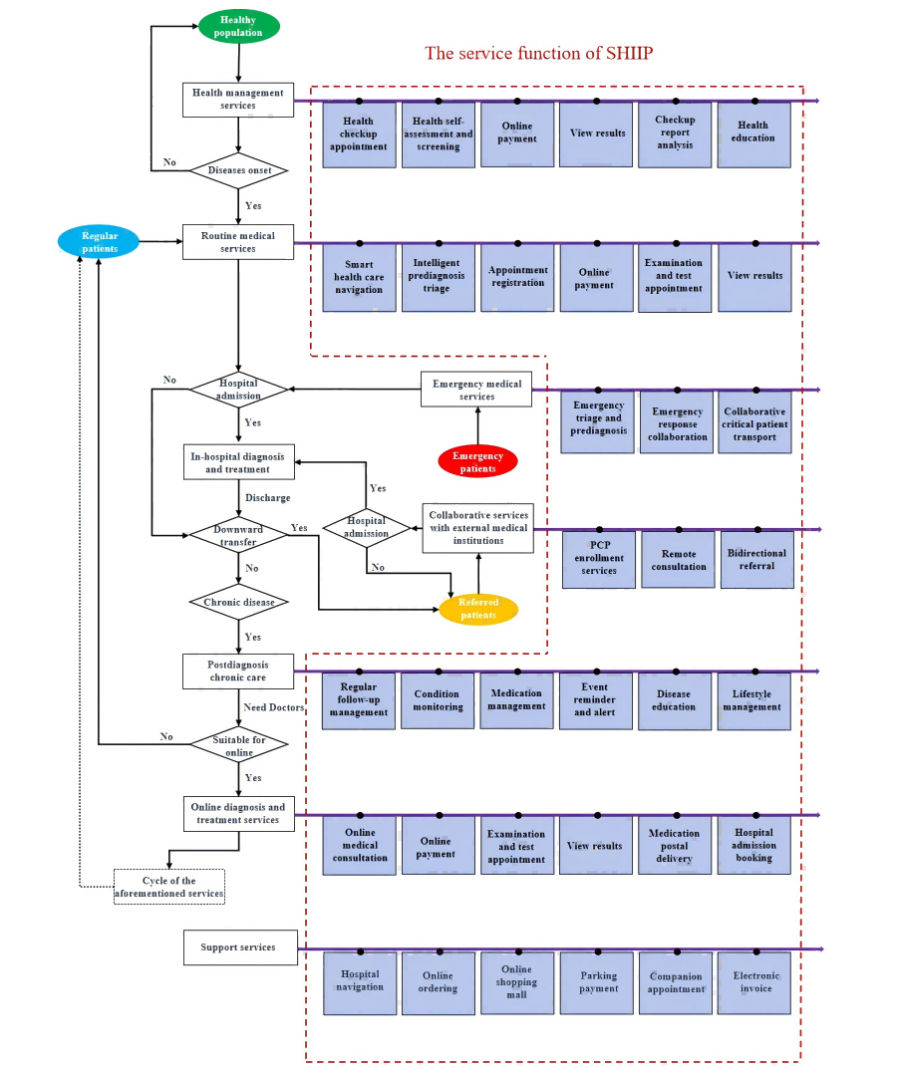
Service processes and functionalities of smart hospital intelligent internet platform for the public. SHIIP: smart hospital intelligent internet platform.

**Figure 2:**
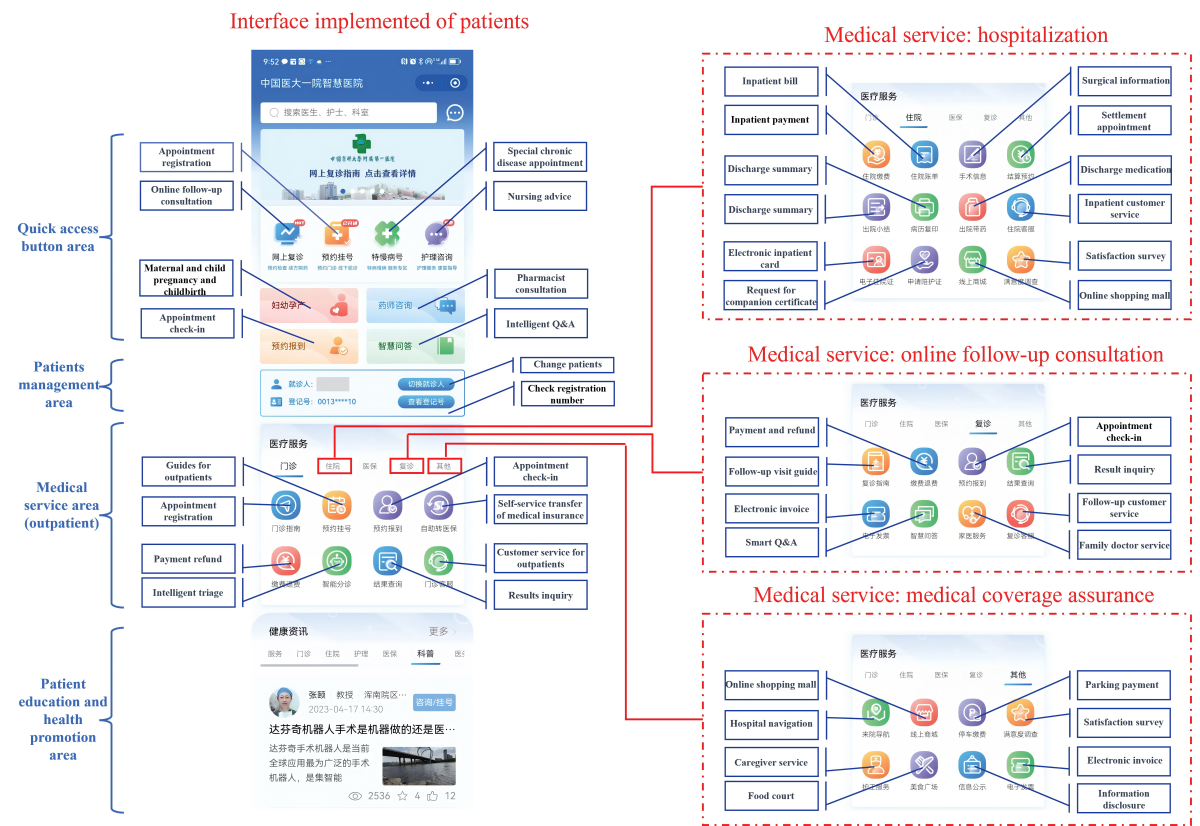
Interface implementation of the public-side. Q&A: question and answer.

First, for the public (as illustrated in Figure 1), the general public is roughly categorized into 4 groups based on their health or medical service requirements, including healthy individuals, routine patients, emergency patients, and referral patients. Addressing the distinct needs of these different groups, SHIIP offers 8 major categories of online services, such as health management services and routine medical services, which also serve as references for the functional partitioning of the interface implementation of the public-side (Figure 2). The ultimate goal is to establish a comprehensive medical and health service ecosystem, encompassing the entire process and lifecycle of patient diagnosis and treatment. Within each service category, numerous specific service projects have been already implemented or are currently in progress, with the platform aiming to continuously expand and introduce additional projects in the future, although Figure 1 provides 3-6 main projects for each service category.

The public involved in the service process is divided into 4 types, each in different colors elliptical box, green for healthy population, red for emergency patients, yellow for referred patients, and blue for regular patients. Starting from the respective colors, the service processes can be identified based on the directions indicated by the arrows (left side), and the corresponding functional items of SHIIP for the public are displayed in the red dashed box (right side). All functions are described in Table S7 in Multimedia Appendix 1.

This figure depicts the home page of SHIIP for the public-side. The functional zones of the home page are described by blue words and black words indicate translation for buttons. The red dashed boxes show the switching pages for 3 medical service objects.

**Figure 3:**
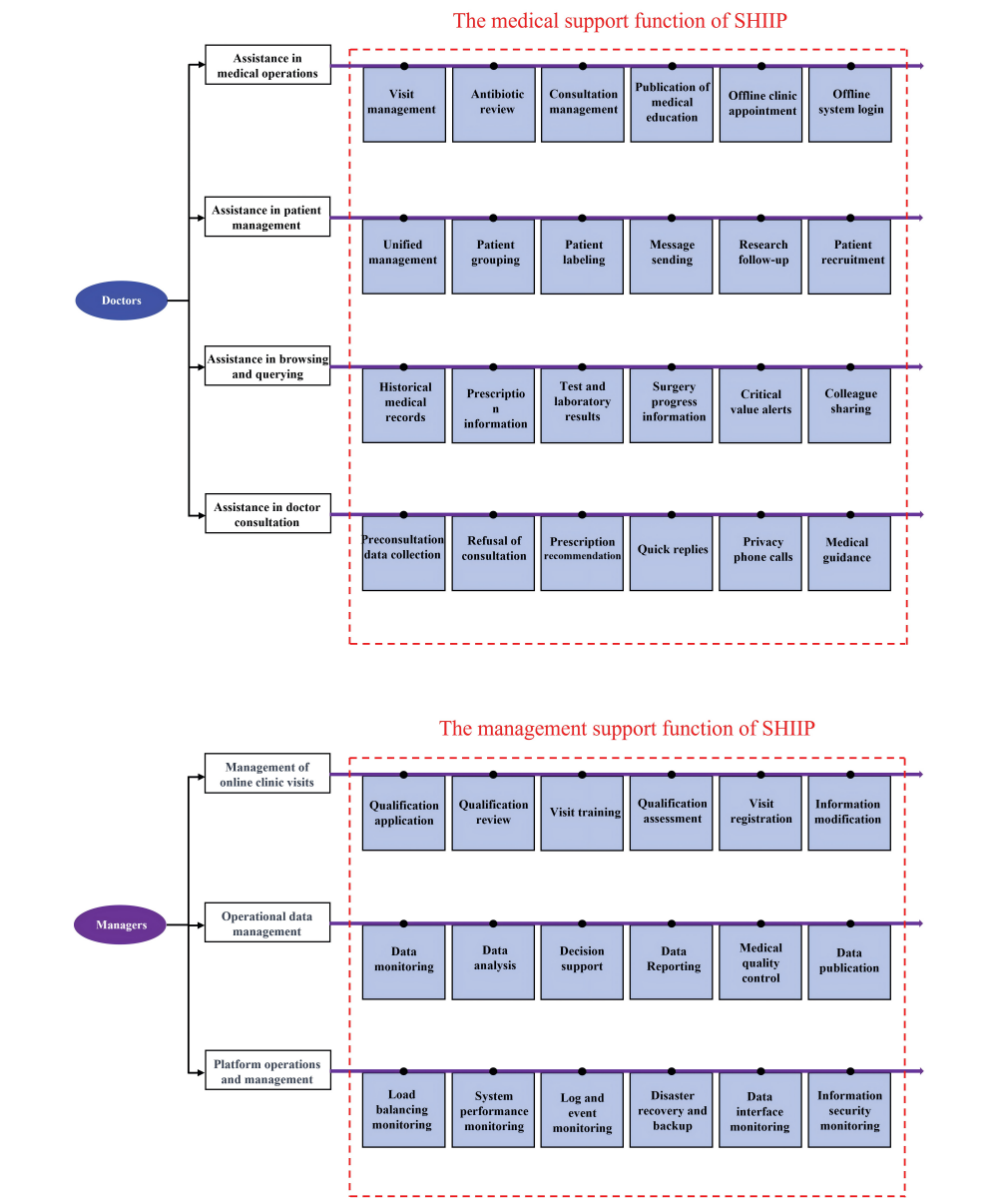
The supporting functionalities for health care workers and administrators. SHIIP: smart hospital intelligent internet platform.

**Figure 4:**
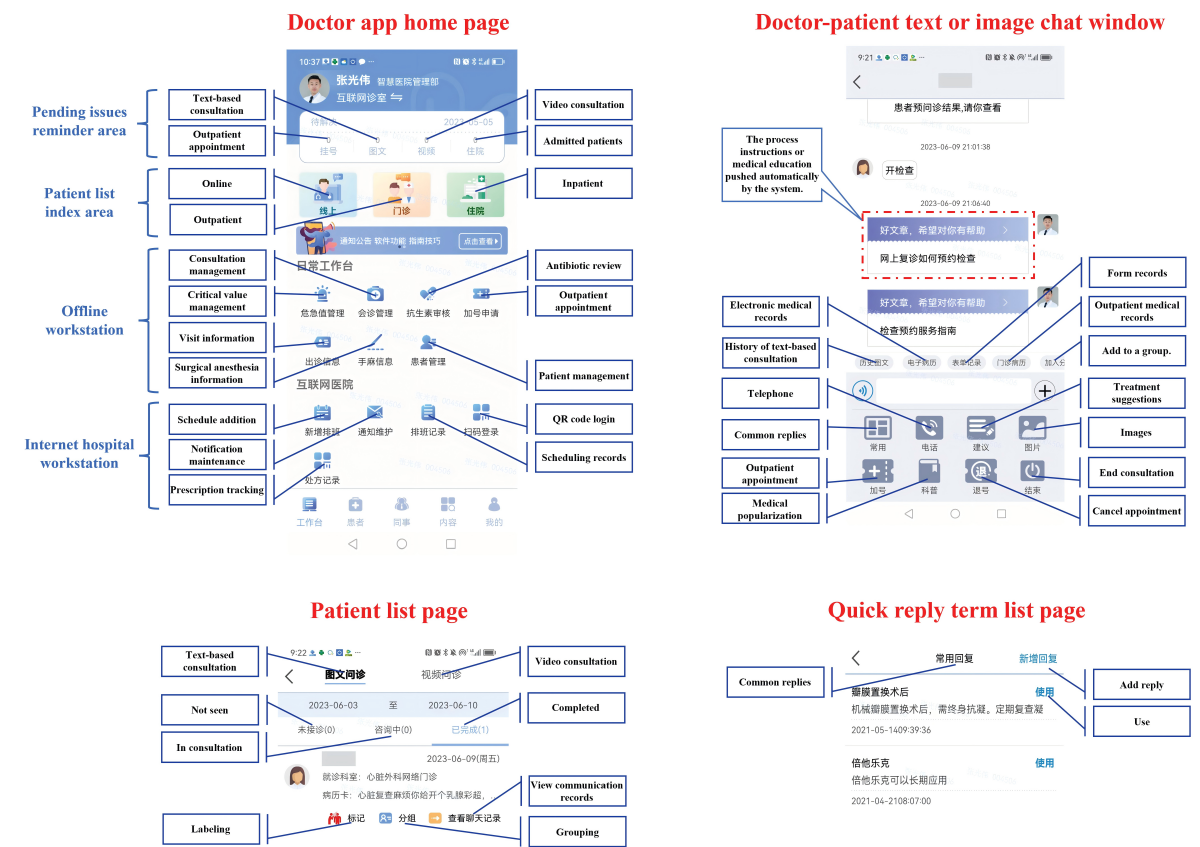
Interface implementation of the health care workers-side.

Second, for health care workers (as shown in Figure 3 and Figure 4), the SHIIP provides comprehensive assistance to doctors in various aspects of their medical practice, enabling them to deliver optimal patient care and streamline their operations, such as (1) assistance in medical operations; the platform supports doctors with visit management, antibiotic review, consultation management, publication of medical education, offline clinic appointment, and offline system login; (2) assistance in patient management; the platform offers unified patient management, patient grouping, patient labeling, message sending, research follow-up, and patient recruitment; (3) assistance in browsing and querying; doctors can access historical medical records, prescription information, test and laboratory results, surgery progress information, receive critical value alerts, and share information with colleagues; and (4) assistance in doctor consultation; the platform facilitates doctor consultations with features such as preconsultation data collection, refusal of consultation, prescription recommendations, quick replies, private phone calls, and medical guidance.

The 2 red dashed boxes represent the main functional items of SHIIP for health care workers and administrators. The functions of health care workers are described in Table S8 in Multimedia Appendix 1. The functions of administrators are described in Table S9 in Multimedia Appendix 1.

The main interfaces of the app for health care workers are shown, including the Doctor app home page, Doctor-patient text or image chat window, Patient list page, and Quick reply term list page (red words). Blue words indicate functional zones and black words present translations for buttons or explanations for regions.

Third, for administrators, the SHIIP offers a range of features and functionalities to support their management responsibilities (Figure 3). These include (1) management of online clinic visits; the SHIIP facilitates the management of online clinic visits through various features, including qualification application, qualification review, visit training, qualification assessment, visit registration, and information modification; (2) operational data management; the SHIIP supports administrators in managing operational data by offering features such as data monitoring, data analysis, decision support, data reporting, medical quality control, and data publication; and (3) platform operations and management; administrators have access to features for platform operations and management, including load balancing monitoring, system performance monitoring, log and event monitoring, disaster recovery and backup, data interface monitoring, and information security monitoring.

### Ethical Considerations

In accordance with the authors’ institutional ethical guidelines, this study does not involve any experiments on human beings or animals, nor does involve any private data that expose personal information about patients. Therefore, it does not seek an ethics review board assessment.

## Results

### Overall Operational Data of the Platform’s Public-Side

**Figure 5:**
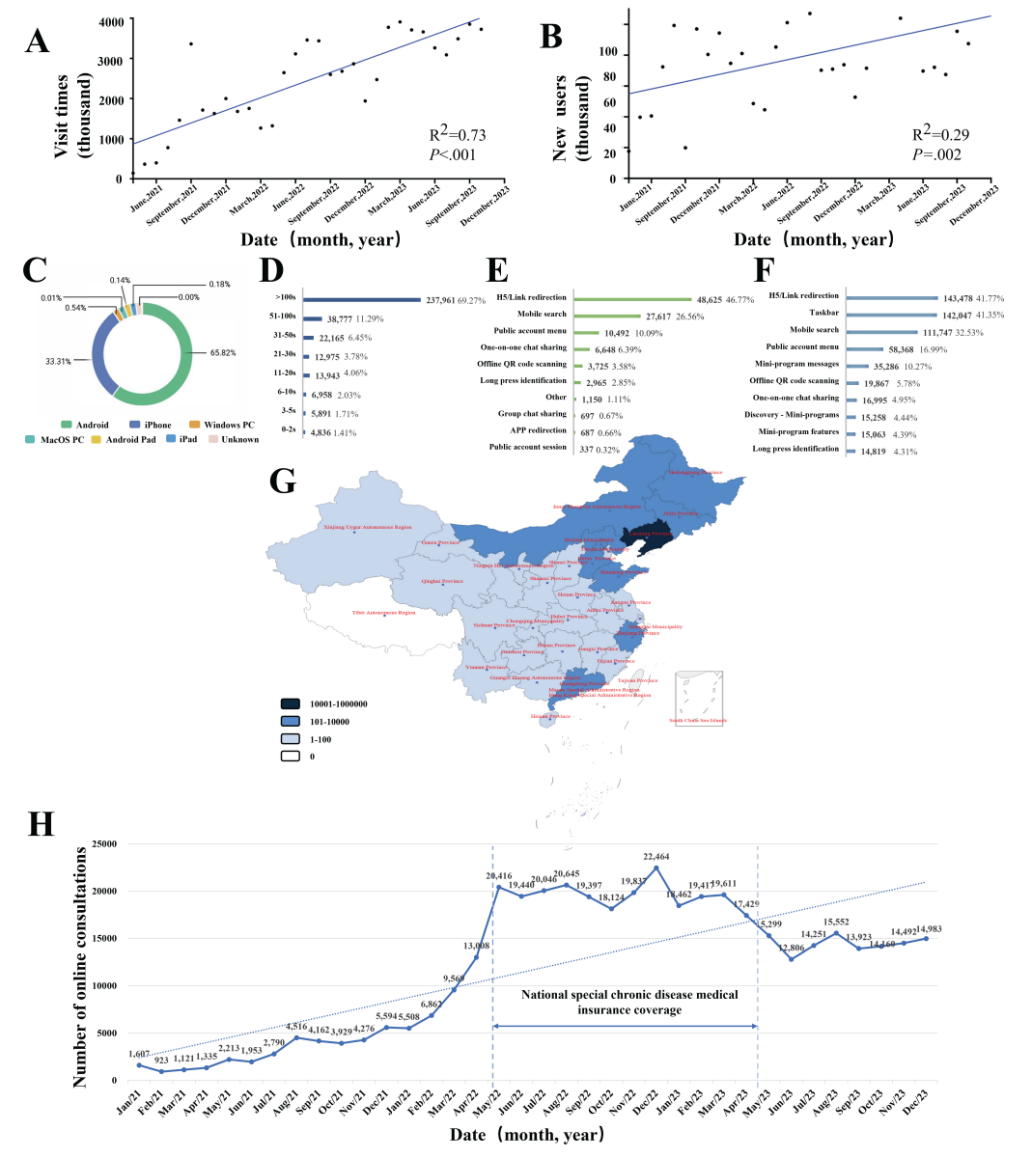
Usage statistics and service data of smart hospital intelligent internet platform.

From its launch until December 31, 2023, the SHIIP recorded a total of 82,279,669 visits. The average user visit frequency was 31, and it accumulated 3.6 million user favorites. Further analysis showed a clear upward trend in both visit volume and new user numbers, with growth rates of approximately 73% (*P*<.001) and 29% (*P*=.002), respectively (Figures 5A and 5B). A device source analysis indicated that over 99% (351,877/354,965) of the visits originated from mobile devices (Figure 5C), with less than 1% (3123/354,965) from desktop computers, indicating smartphones as the predominant means of accessing the IH services. Temporal analysis of platform visits showed that over 95% (325,821/343,506) lasted for more than 10 seconds, and nearly 70% (237,961/343,506) exceeded 100 seconds (Figure 5D), identifying a majority of meaningful engagements. Further analysis of the sources of new patient user traffic revealed that the 5 primary methods are link redirection, mobile search, public account menu, one-on-one WeChat (Tencent Holdings Limited) sharing, and offline QR code scanning (Figure 5E). The foremost 3 of these methods, combined with taskbar and miniprogram messages, also emerged as the principal sources of system access (Figure 5F). These findings underscore the significance of the 5 identified pathways for the analysis, development, and managerial decision-making of the SHIIP platform, delineating critical user traffic trends essential for optimizing engagement strategies.

It presents a comprehensive overview of the SHIIP’s usage, user growth, drug delivery distribution, traffic sources, access channels, user engagement duration, and platform accessibility. The growth trend of the number of visits to the platform is shown in Figure 5A. The continuous growth in new patient user numbers over time is displayed in Figure 5B. Figures 5C-5F show, respectively, the traffic diversion sources of new users, distribution of access sources for the platform, distribution of users’ access duration, and the distribution of platforms accessed by users. Figure 5G showcases the visual representation of the distribution and volume of medicine deliveries across different regions and darker blue indicates more medicine delivery orders in the area. Figure 5H shows the curve of consultation volume.

### The Overview of Online Consultations

Under the autonomous decision-making mechanism for online services, 574 doctors (out of 797), representing 72% of all qualified physicians, engaged in online consultations, signifying substantial acceptance of online medical practices among the physician community. These practitioners spanned 64 departments and represented 44 distinct medical subdisciplines, encompassing 96% (64/66) and 94% (44/47) of all medical departments and subdisciplines in our hospital, respectively. Although there was an uneven distribution among departments and subdisciplines, with disparities such as 22 doctors in cardiovascular medicine compared with only 1 in general geriatric (Table S4 in Multimedia Appendix 1), the extensive participation across diverse medical fields and specialties has been fundamental in enabling the platform to support a significant volume of medical services throughout its 3 years of operation. The number of online medical consultations reached 420,120 from 180,020 patients, with 124,422 electronic prescriptions, 24,862 examination and test orders, and 92,285 medication deliveries. The delivery range covered 30 provinces, with the farthest delivery distance exceeding 3500 kilometers, demonstrating truly nationwide services (Figure 5G). The total volume of refusals was 64,306, constituting an overall refusal rate of 16%. The primary reason for these refusals was the physicians’ assessments that the patients’ conditions were unsuitable for online consultations. A quantitative analysis of online medical consultations indicates a consistent upward trend, except for a significant fluctuation between April 2022 and May 2023 (Figure 5H), which was explicitly affected by health insurance policies and pandemic management measures. This underscores the critical influence of policy factors on the development of IH.

### The Stratified Analysis of Online Consultations

The annual variations in consultation and refusal volumes across different departments are summarized in Table S3 in Multimedia Appendix 1. Breast surgery I, gastroenterology, rheumatology and immunology, endocrinology, and cardiovascular emerged as the top 5 departments, with total consultation numbers of 26,636, 28,039, 24,152, 18,984, and 16,407, respectively. Furthermore, many departments exhibited a year-over-year growth trend consistent with the overall trajectory, such as endocrinology (increasing from 1417 in 2021 to 14,147 in 2023) and thoracic surgery (from 1117 in 2021 to 11,676 in 2023). Conversely, some departments experienced a decline, for instance, cardiovascular medicine (from 333 in 2021 to 122 in 2023) and endocrinology (from 44 in 2021 to 22 in 2023). Similar patterns of change were also observed at the level of subdisciplines (Table S5 in Multimedia Appendix 1). Beyond policy factors, the reasons for these changes warrant further analysis, which could contribute to regulating departmental consultation volumes.

Furthermore, the analysis at the physician level revealed an average annual consultation volume of approximately 189 per doctor, with an average annual patient-to-doctor ratio of about 1:3. However, significant disparities in this ratio were observed across different subdisciplines, with the largest being 1:1654 in traditional Chinese medicine in 2023, and the smallest at 1:2 in rehabilitation medicine and geriatrics in the same year. In addition, certain departments experienced considerable fluctuations in their annual patient-to-doctor ratios, such as traditional Chinese medicine, which shifted from 1:4 in 2021 to 1:295 in 2022, and then to 1:1654 in 2023. These findings highlight 2 critical insights. First, the development of our IH remains uneven, with many departments needing enhancement; and second, some departments or subdisciplines have already demonstrated strong capabilities in internet-based medical services, affirming the significant role and potential for growth of IHs.

### The Disease Spectrum Analysis of Online Consultations

In our disease spectrum analysis, we identified 3191 diseases involved in online consultations, constituting 8.86% (3191/36,016) of all diagnoses currently used in our hospital. The most prevalent condition was health check-ups (n=39,361), followed by common and chronic internal medicine conditions such as nodular goiter (n=6112) and coronary atherosclerotic heart disease (n=3544). Notably, there were also many surgical conditions, including abdominal pain (n=3443) and breast malignancy (n=3190), and even emergency conditions in their chronic phase, like hypokalemia (n=71) and acute pancreatitis (n=31), demonstrating a promising capacity of online consultations for broad disease coverage. A comparative analysis with traditional in-person outpatient data for the top 20 conditions treated (Table S2 in Multimedia Appendix 1) revealed that more than 10% (17,301/126,235) of patients, for certain diseases, opted for online consultations, with the highest being chronic hepatitis B (1523/5919, 25.73%) and systemic lupus erythematosus (1418/5636, 25.16%), followed by breast malignancy (3190/21,961, 14.53%), thyroid malignancy (3118/23,054, 13.52%), anxiety state (2614/24,447, 10.69%), and pregnancy (1712/16,292, 10.51%). However, common conditions like diabetes mellitus (1935/72,378, 2.67%) and hypertension (1890/110,401, 1.71%) showed lower online consultation rates. These results not only reveal a development imbalance similar to that observed at the departmental and subspecialty levels but also suggest that over 25% of patients could be adequately managed through IH-derived services.

### Smart Hospital Intelligent Internet Platform Support for Traditional Offline Services

Beyond online consultations, SHIIP has conducted a total of 6,965,566 prediagnosis triages, encompassing 5,895,867 outpatient, 689,576 emergency, 324,271 internet medical, 50,471 fever clinic, and 5381 artificial intelligence (AI) screenings for COVID-19. Over the past 3 years, SHIIP has facilitated 4,995,835 offline outpatient appointments, accounting for 84% (4,995,835/5,928,673) of all bookings, with a notable increasing trend from 67% (1,431,544/2,135,093) in 2021 to 92% (1,483,753/1,606,790) in 2022 and reaching 95% (2,080,538/2,186,790) in 2023. Detailed annual appointment data by department are summarized in Table S6 in Multimedia Appendix 1. In addition, the total number of online examination and test appointments was 187,356. There were 9617 cases of collaborative emergency assistance. In terms of remote consultations, 1311 cases of multidisciplinary remote consultations were completed. Chronic disease services were provided to 55,148 individuals. In terms of health education, 113 online live consultation sessions were conducted, with 2025 medical education videos and articles published, and a total of 15,148,310 views. There were 2711 appointments for physical examinations, with 486 interpretations of physical examination results. In terms of smart support services, 329,704 orders were completed for online delivery of life support products, 246,677 copies of electronic medical records were printed, and 328,111 applications were made for electronic hospitalization support cards.

## Discussion

### Principal Results

Despite the widespread attention to IHs, there are absent comprehensive and detailed investigations of their platforms. This study presents a pioneering single-center experience, offering a comprehensive and systematic exposition of the development of IHs within the framework of a “Trinity” smart hospital for the first time [1,7-9]. It encompasses diverse dimensions, including platform architecture design, technological applications, service content and processes for the public, auxiliary functionalities for health care professionals, management tools for administrators, and corresponding operational data and outcomes. Our SHIIP diverges significantly from the narrow perception held by many individuals, who merely associate IHs with the provision of online diagnosis and drug delivery [13]. Through practical implementation, we have demonstrated a new model for the construction and management of IHs, which provides concrete examples to elucidate the conceptual definition, functional positioning, and future development direction of IHs.

Our platform has presented a remarkable set of data on online diagnosis and treatment, medication delivery, remote consultations, and health consultations, demonstrating the significant role of an IH as an indispensable component of our physical hospital services. These exciting results have provided robust evidence of the efficacy of our IH construction and management paradigm, substantiating the viability of health care institutions developing IHs. This success not only validates the operational model but also establishes a benchmark for the strategic integration of digital and traditional health care services. Our data indicate that online consultations encompass a vast majority of departments and prevalent illnesses, suggesting that telemedicine is broadly applicable at the disease level. Furthermore, numerous departments and specialties within our hospital have significant potential for growth in their online consultation ratios, heralding substantial prospects for the development of internet-based health care facilities. More importantly, a consistent increase in monthly traffic and the number of new users indicates a progressive rise in public acceptance and recognition of online health care services, which have also gained widespread recognition from most doctors. These growing trends suggest that IHs will play an increasingly important role in the health care landscape [4,14].

In addition, benefiting from the intelligent and user-friendly features on both the patient and physician sides, it takes an average of only 6 minutes to complete an online medical visit on the platform, significantly shorter than the time required for traditional outpatient visits involving transportation, waiting, consultation, and medication retrieval. Combining these actual business data of our platform and the subjective reports collected from users through surveys conducted by Zhejiang Hospital [15], there is an unequivocal conclusion that IHs will undoubtedly bring convenience and improve work efficiency for users, which stands as a key determinant for the limitless growth potential of IHs in the future. In fact, the internet is precipitating a global transformation in health care service models. In Europe, some countries, like Sweden, have integrated telehealth into their national health system, resulting in significant improvements in health care efficiency and patient satisfaction [16,17]. In the United States, some IHs have elevated health care standards through innovative technological efforts [18]. In New England, the exploration of digital health technologies, particularly the lens of telemedicine’s feasibility for patients, provides critical insights into the complexities of digital health integration and the universal challenges faced in ensuring the efficacy and acceptance of such technologies [19]. Therefore, IHs are emerging as a critically important new model in medical services, poised to reshape the landscape of health care delivery globally.

A large amount of noninternet medical data on the platform, including outpatient appointments, result viewing, and online shopping, signify the deep integration of our IH with the physical hospital business. Drawing from similar successful experiences in other hospitals [9,20], it is evident that the digitization of traditional medical services and their incorporation into IH platforms is not only feasible but also highly significant, even essential. This assertion has also been well-supported by the forementioned analogous international examples [21,22]. Furthermore, IHs have showcased their significant potential in prevention and control against the COVID-19 pandemic [3], which is confirmed by our substantial business data again, including online consultations, intelligent screening, and prediagnosis of fever clinics. Therefore, they are poised to play a crucial role in future responses to similar unforeseen events in the realm of public health [6]. Furthermore, it is indicated that our internet platform has become a crucial avenue for delivering health education and promoting public education and awareness, underscoring the important role of IHs in improving the health literacy of residents. Recently, several Chinese hospitals have also reported outstanding achievements in various aspects of their respective IHs. These encouraging results demonstrate the increasingly vital role of IHs in China’s health care service system, and the upward trends in our multiple data metrics suggest a promising outlook for its sustained and positive development in the future.

Our use data indicate that smartphones have become the mainstream device for accessing IH services, emphasizing the need for the platform to prioritize the development of mobile apps to meet the demands of primary users. WeChat and Alipay (Alibaba group), each with over 1 billion users, are widely used mobile social media and mobile payment platforms in China. They have emerged as common support systems for building IH platforms, due to their convenience, flexible development frameworks, diverse development technologies, open interfaces, unified identity authentication, and secure reliability [23,24]. Within their frameworks, the SHIIP was designed to provide services through various forms for catering to the diverse user preferences and usage habits, such as miniprograms and web pages, resulting in significant recognition and usage from a large number of users. Similar outcomes were achieved by multiple IHs, such as Sichuan University West China Second University Hospital [25], providing strong evidence that this strategy will become the mainstream approach for the development of IHs.

### Innovation in Concepts, Methods, and Technologies

Unlike traditional physical hospitals, the capabilities of IHs are directly determined by the functionality of information platforms, as their operations rely entirely on the latter [9]. Regarding this aspect, various medical institutions have been actively exploring, from the Second People’s Hospital of Guangdong Province sponsoring the first public internet medical platform (Guangdong IH) for online consultation [1], to the First Affiliated Hospital of Zhejiang University attempting family physician services and management of chronic diseases based on online diagnosis and treatment platform [9,26], followed by many ones exploring further various functionalities in terms of patient services or assistance for health care professionals and managers [8,10,12,27]. Until recently, it has been proposed by the Affiliated Second Hospital of Zhejiang University School of Medicine that the IH platform has begun to integrate partially and irreversibly into traditional health care systems [4]. This progressive evolution has led to a clearer and more refined understanding of their functionality and construction direction, serving as a valuable reference for platform development. Furthermore, the success of various third-party commercial IH platforms with their highly flexible and practical features, including “Ping An Good Doctor,” “Haodaifu Online,” “Jingdong Health,” and others [8,28], has also provided invaluable experience for the establishment of IH platforms in public health care institutions.

Combining these experiences and the aforementioned novel theories in this field, we have developed the comprehensive SHIIP, which incorporates several innovative features crucial to its functionality. First, by actively expanding online services and digitalizing traditional health care as illustrated in Figure 2, it further enhances the “patient-centered” service model, standing out with greater comprehensiveness and systematization compared to previously reported online medical platforms [29]. The aim is to minimize time and financial burdens benefiting from a maximally convenient approach that relies on the internet to overcome time and geographic constraints [7,27], such as “single visit” or “zero visit” seeking medical care, an impenetrable challenge for traditional offline outpatient services in China. Second, it serves as an efficient tool for health care professionals and managers, embodying the balanced development of the “Trinity”. As summarized in Figure 3, the platform not only incorporates diverse, convenient, flexible, and highly practical functionalities to assist doctors in their work but also continuously achieves digitization in management, which will contribute to the improvement of work and management efficiency [30]. Third, it establishes a data platform that facilitates data interoperability among systems through various means, such as data interface transmission, supporting our platform as a central hub for integrating online and offline functionalities. This innovative approach is expected to further promote the deeper integration of online and offline health care services, building upon existing reports on IHs [4,8], to ensure a smooth flow of health care business processes. Furthermore, based on its ability of data and health care business interoperability, our SHIIP has acted as a pioneer of comprehensive health care services mode throughout the entire process and lifecycle, attempting to cover various health care needs from various patients at various situations as shown in Figure 1. It presents a dramatic extension in the availability of services beyond traditional offline settings, although there are so many aspects that need to be improved.

Due to the complexity and comprehensiveness of our IHs, there is a tremendous demand for information platform development, comparable to the informatization efforts of traditional physical hospitals. Accomplishing this formidable task solely relying on a single system or software vendor is nearly impossible, similar to how the information systems of traditional physical hospitals require hospital information systems, picture archiving and communication systems, laboratory information systems, and other systems from different software vendors. Our platform currently integrates 16 systems from 9 software vendors, which were built before, during, or after the implementation of the internet diagnosis and treatment system, leading to a complex and extensive project in the construction of SHIIP. Therefore, meticulous considerations are crucial for overall planning, including functional coordination, goal achievement, technological advancement, anticipated iterative updates, system scalability, integration, and server allocation. The technological roadmap cannot be achieved instantly, necessitating a step-by-step construction or transformation approach, gradually advancing the integration strategy through various means. In fact, some other IHs have already validated the feasibility of this modular construction approach [4,8]. However, our exploratory work lacks traces and successful cases for reference in many aspects, necessitating continuous adjustments and revisions, despite meticulous planning. This platform construction experience emphasizes that the development of IH information systems should be a process characterized by comprehensiveness, planning, complexity, and continuity, extending far beyond the establishment of a diagnosis and treatment system. Considering that each hospital has different foundational conditions, we recommend formulating strategies tailored to their own characteristics based on the existing state of informatization when constructing IHs.

### Data Privacy and Security

Throughout the SHIIP building process, we were fully aware that data privacy and security were our top priorities. Specifically, we have implemented advanced technologies and measures in information security. Our measures include hiding sensitive information, encrypting personal and medical data, and using firewalls, authentication, and security audits to strengthen our defenses. Furthermore, our database servers are located within a core network, isolated from external networks, providing additional protection against unauthorized access and cyberattacks. Despite facing more than 10 cyber threats daily, our strict security protocols have prevented data breaches. This demonstrates our commitment to information security and ensures our platform integrates online and offline functions without compromising data privacy.

### Policies and Norms

In navigating the complex landscape of IHs, adherence to and alignment with established policies and standards have been paramount. As delineated in Table S1 in Multimedia Appendix 1, key regulations such as the “Administration Regulations on Internet Diagnoses and Treatments (Trial)” and “Administration Regulations on Internet Hospital (Trial)” have provided a foundational legal framework that has informed our platform’s development, ensuring compliance with quality and safety standards for online diagnoses and treatments. Furthermore, the “Smart Service Scoring System” and “Grading Evaluation Standard for The Application Level of Electronic Medical Record System” have served as benchmarks for assessing the integration of medical resources and the application level of our electronic medical records systems, respectively. These standards not only guided the technological development of our SHIIP but also ensured that our services aligned with the expectations for smart services for the public, smart medicine for medical workers, and smart management for hospital administrators. The “Smart Management Scoring System,” in particular, has been instrumental in evaluating the effectiveness of our management practices, reflecting our commitment to maintaining a high standard of smart management. The convergence of these policies and standards has been instrumental in shaping a conducive operational environment for our SHIIP, underscoring the pivotal role of regulatory frameworks in fostering innovation while ensuring patient safety and service quality.

### Development Planning and Prospect

In the future, the platform will continue to adhere to the concept of continuous optimization and improvement through the adoption of advanced technologies, aiming to better serve patients, doctors, and administrators. First, AI technology is recognized as playing a crucial role in health care, especially with the emergence of generative AI models like ChatGPT (OpenAI) [31,32]. Building upon the existing AI apps, our platform plans to further expand the scope and methods of AI use, making it more intelligent, personalized, and seamless. Second, extensive research has revealed that the application of medical Internet of Things (IoT) devices is another important direction for enhancing the functionality and capabilities of health care information platforms [33]. In the next phase, the platform intends to gradually integrate IoT devices such as blood pressure monitors and glucose meters through data interface connections, achieving a more comprehensive IoT integration. In addition, the platform will leverage blockchain technology to further enhance information security, as it is considered an effective means to ensure secure sharing and interaction of medical data [34]. Furthermore, innovative health care models like virtual wards and remote surgeries have shown vast potential and will be areas of exploration for our platform [35].

### Limitations

Despite the progressive strides taken by our SHIIP, it remains in an embryonic phase of development, characterized by pronounced imbalances in the breadth of services provided, the engagement of various medical departments and professionals, and the spectrum of diseases covered. The foundational structure and administrative frameworks of the SHIIP are yet to be optimized, reflecting a need for ongoing refinement. Furthermore, the lack of comprehensive online quality control protocols tailored to specific ailments underscores a gap in our current operational model. This deficiency points to the imperative for methodical advancements and the establishment of stringent quality assurance measures in future endeavors to ensure the delivery of high-caliber online health care services.

### Conclusions

The theoretical framework of IHs in China has undergone significant advancements, with our practical implementation providing substantial evidence of its feasibility and innovation. The extensive construction experience and operational effectiveness benefited from SHIIP, strongly underscore the pivotal role of information platforms in the development of IHs, and their significance in traditional physical hospitals as well. Therefore, it is very important to select an appropriate construction model. While certain aspects still require further validation and refinement, our platform has already achieved considerable scale and will continue to undergo continuous optimization and enhancement. This pioneering exploration holds tremendous significance and serves as a valuable guiding reference for IHs construction and the progressive development of the internet health care sector.

## Appendix

Multimedia Appendix 1 Additional files.

## Abbreviations

AI: artificial intelligence
IH: internet hospital
IoT: Internet of Things
SHIIP: smart hospital intelligent internet platform
