# Supplementary material for: In-Depth Examination of the Functionality and Performance of the Internet Hospital Information Platform: Development and Usability Study
Source: J Med Internet Res. 2024 Nov 8;26:e54018. doi: 10.2196/54018 (PMC11584526; doi:10.2196/54018)
Supplement: Multimedia Appendix 1 [file jmir_v26i1e54018_app1.docx]

**Objective and Concept:** The primary objective of constructing this platform is to support the operation of internet hospitals. Another aim involves in enhancing the service capabilities of the traditional healthcare system by innovating service content and expanding the scope of services. Furthermore, it seeks to improve the level of smart hospital construction, thereby promoting comprehensive and high-quality development of hospitals. Additionally, it explores successful construction and operation models for internet hospitals and validates the latest theoretical frameworks through practical implementation. According to the latest advancements in the field, the platform supporting the IHs operation should possess the following functionalities. Besides facilitating online diagnosis and treatment, it should also achieve a seamless integration of online and offline healthcare services, ensuring consistent care throughout the patient journey, covering the entire lifecycle of prevention, treatment, and long-term disease management. Furthermore, it should aim to enhance the health literacy and self-management capabilities of individuals. Additionally, while providing the aforementioned "smart services" to patients, it should leverage advanced information technology to enable "smart healthcare" for assisting healthcare professionals and "smart management" to support administrators.

**Policy Support:** The development and operational strategies of internet hospitals, as well as the platform, are supported by policies such as the "Administration Regulations on Internet Diagnoses and Treatments [Trial]", "Administration Regulations on Internet Hospital [Trial]", and "Administration Specifications for Telemedicine Services [Trial]". These policies aim to regulate and guide the functioning of SHIIP, ensuring quality and safety in online medical services, and supporting the integration of smart hospital concepts. (Table S1).

**Blueprint of the SHIIP:** The SHIIP with the diagnosis and treatment platform as its core, currently integrates and consolidates 15 business systems, as presented in the overall framework (Figure S1). In the future, additional systems or functionalities will be incorporated continuously to meet the evolving needs in this field and further enhance the platform. The servers deployed in the platform are divided into two areas: the Core Area and the Buffer Area. The platform has two types of network connections. One is a dual-vendor public service network dedicated to Internet hospital services, and the other is a dedicated medical line network for data transmission between medical institutions and regulatory platforms. They refer the foundational support system (including technological support, service support, and management support), the standard and regulatory system (comprising legal regulations, industry standards, technical standards, medical standards, and data standards), and the assurance system (encompassing data security, quality assurance, safety measures, service assurance, and risk management). There are four types of applications as followed: 1) The application program on smartphones enables patients (the general public) to receive internet hospital services through mobile devices. 2) The application program on smartphones facilitates healthcare professionals to perform their work on the internet hospital platform using mobile devices. 3) The desktop application program on PCs allows healthcare professionals to work on the internet hospital platform in fixed locations. 4) The desktop application program on PCs enables managers to administer the internet hospital platform in fixed locations.

**Technical Architecture:** To meet specific technical requirements such as stability, advanced capabilities, openness, scalability, and maintainability, the platform's technical architecture was divided into five layers: presentation layer, access layer, application layer, middleware layer, and storage layer. The presentation layer consisted of a PC browser website for staffs and managers, WeChat Official Account (H5) and WeChat Mini Program for the public, as well as native APP for staffs. The access layer comprised an API gateway that provides service invocation and bus service scheduling using protocols such as HTTPS, WebSocket, and WebRTC. The application layer implemented various business scenarios including appointment registration, in-clinic payments, examination scheduling, online consultations, and medication delivery. The middleware layer provided reusable common capabilities, including user middleware, payment middleware, messaging middleware, and instant messaging middleware. The storage layer included non-persistent storage (e.g., Redis) and persistent storage (e.g., MySQL and cache database). Middleware products included message queues, logging and search engines, resource monitoring, and monitoring visualization. The service governance component encompassed service rate limiting, registration, circuit breakers, load balancing, monitoring, alerting, log management, and container services. The technology stack involved programming languages and frameworks such as Vue, jQuery, weUI, vant, and MpVue for the presentation layer; Java, AndroidX, SQLite, Swift, Objective-C, and UIKit for the native app; PHP, Symfony, Swoole, and M language for the application layer; and PHP, Yaf, Hyperf, and Node.js for the middleware layer. (Figure S2)

**Network Structure:** The network served as the foundation for the platform's construction. It provided the framework and connectivity required for the platform to function effectively. The network facilitated the transmission of data, communication between different components of the platform, and seamless interaction between users and the platform's services. A robust and reliable network architecture was essential to ensure the platform's stability, scalability, and overall performance. The deployment of various servers at different network levels was illustrated in the network topology logical structure diagram (Figure S3).

**Data Service Architecture:** To achieve data collection, transformation, uploading, monitoring, archiving, and data services, as well as enable data interoperability, the platform's data service system architecture was designed with a messaging mechanism as the technical core. The architecture included the following components: 1) Data Integration: Supports integration with multiple technologies through pre-built adapters (. NET and PHP) and various communication protocols (TCP, HTTP, Socket). 2) Performance Monitor: Monitors key metrics, provides data integration capabilities with big data platforms, and supports multiple transmission protocols. It facilitates interface transformation and data format conversion. It also provides runtime monitoring functionality with progress display and detailed logging. 3) Data Collection: Supports both real-time and non-real-time data exchange, ensuring secure and reliable data transfer in both upstream and downstream directions. It supports batch and case-based real-time data exchange modes, as well as incremental data collection. The architecture is designed to be flexible for future interface changes, additions, and extensions. It enables concurrent data collection from multiple data sources. (Figure S4).

**Table S1.** Policies and Standards in the IH Landscape

| Policy/Standard | Description |
| --- | --- |
| Administration Regulations on Internet Diagnoses and Treatments [Trial] | Regulates internet medical services, outlining quality and safety standards for online diagnoses and treatments. |
| Administration Regulations on Internet Hospital [Trial] | Governs the operation of internet hospitals, detailing functional, service, and safety requirements. |
| Administration Specifications for Telemedicine Services [Trial] | Provides a framework for telemedicine services, setting technical and quality standards for consistency and reliability. |
| Smart Service Scoring System | Evaluates smart services for the public, assessing the integration of medical resources, service optimization, and technological convenience. |
| Grading Evaluation Standard for The Application Level of Electronic Medical Record System | Focuses on medical workers, grading the application level of EMR systems to standardize diagnosis, treatment, and support decision-making. |
| Smart Management Scoring System | Targets hospital administrators, evaluating the efficiency and effectiveness of smart management practices and technological integration. |

**Table S2.** Traditional In-Person Outpatient Services vs. Online Consultations of the Top 20 Conditions Treated

| Rank | Primary Diagnosis | IHs | Offline |
| --- | --- | --- | --- |
| 1 | Health Checkup | 39361(7.77%) | 466892(92.93%) |
| 2 | Nodular Goiter | 6112(6.72%) | 84897(93.28%) |
| 3 | Coronary Atherosclerotic Heart Disease | 3544(5.84%) | 57111(94.16%) |
| 4 | Abdominal Pain | 3443(2.70%) | 124155(97.30%) |
| 5 | Breast Malignant Tumor | 3190(14.53%) | 18771(85.47%) |
| 6 | Breast Hyperplasia | 3129(4.17%) | 71883(95.83%) |
| 7 | Thyroid Malignant Tumor | 3118(13.52%) | 19936(86.48%) |
| 8 | Breast Lump | 2831(5.84%) | 45669(94.16%) |
| 9 | Anxiety State | 2614(10.69%) | 21833(89.31%) |
| 10 | Hyperthyroidism | 2275(11.46%) | 17575(88.54%) |
| 11 | Arthralgia (Joint Pain) | 2113(2.20%) | 93817(97.80%) |
| 12 | Diabetes Mellitus | 1935(2.67%) | 70443(97.33%) |
| 13 | Gastritis | 1920(3.05%) | 61079(96.59%) |
| 14 | Hypertension | 1890(1.71%) | 108511(98.29%) |
| 15 | Pregnancy | 1712(10.51%) | 14580(89.49%) |
| 16 | Lung Shadow | 1709(2.89%) | 57515(97.11%) |
| 17 | Thyroid Lump | 1571(1.52%) | 101501(98.48%) |
| 18 | Chronic Hepatitis B | 1523(25.73%) | 4396(74.27%) |
| 19 | Rheumatoid Arthritis | 1451(15.99%) | 7625(84.01%) |
| 20 | Systemic Lupus Erythematosus | 1418(25.16%) | 4218(74.84%) |

**Table S3.** The percentage of each department's consultation volume relative to its total online visit volume over the past three years (with a rejection rate being its complement).

| Department | 2021 | 2022 | 2023 |
| --- | --- | --- | --- |
| Breast Surgery I | 62.67% | 71.54% | 81.54% |
| Gastroenterology | 94.11% | 93.15% | 90.21% |
| Rheumatology and Immunology | 95.68% | 92.80% | 89.16% |
| Endocrinology | 76.39% | 64.72% | 84.71% |
| Traditional Chinese Medicine | 91.67% | 93.52% | 94.17% |
| Cardiovascular Medicine | 91.22% | 57.19% | 84% |
| Thoracic Surgery | 84.18% | 74.10% | 75.83% |
| Urology | 73.34% | 81.26% | 76.55% |
| Psychology | —— | 84.22% | 89.45% |
| Gynecology | 83.31% | 82.31% | 70.92% |
| Infectious Diseases | —— | 89.16% | 92.33% |
| Thyroid Surgery | 82.35% | 76.51% | 76.53% |
| Hematology | 82.95% | 85.19% | 87.52% |
| Vascular and Thyroid Surgery | 13.51% | 79.29% | 82.01% |
| Neurology | 92.75% | 86.45% | 81.55% |
| Nephrology | 75.56% | 81.75% | 86.44% |
| Plastic Surgery | 93.06% | 89.30% | 86.41% |
| Pediatrics | 34.09% | 93.19% | 92.64% |
| Breast Surgery III | 89.92% | 77.42% | 77.72% |
| Oncology | 76.42% | 85.77% | 66.28% |
| Otorhinolaryngology | 63.31% | 80.58% | 75.04% |
| Hepatobiliary Surgery | 80.93% | 85.55% | 86.18% |
| Breast Surgery II | 92.80% | 84.21% | 85.18% |
| Nuclear Medicine | 88.94% | 86.37% | 87.25% |
| Oncology II | —— | 83.58% | 86.40% |
| Obstetrics | 86.25% | 83.26% | 76.28% |
| Thoracic Surgery II | 86.17% | 78.73% | 86.94% |
| Care Outpatient | 96.77% | 86.37% | 71.37% |
| Neurosurgery | 74.73% | 67.83% | 70.80% |
| Ultrasound Diagnostics | 96.77% | 83.86% | 75.94% |
| Geriatric Respiratory and Infectious Diseases | 94.44% | 74.71% | 79.48% |
| General Medicine | 81.59% | 83.67% | 76.55% |
| Pain Medicine | 96.33% | 92.77% | 89.29% |
| Orthopedics | 91.30% | 73.87% | 43.03% |
| Cardiac Surgery | 72.34% | 90.42% | 82.87% |
| Pharmacy Services | —— | —— | 87.85% |
| Interventional Medicine | 80% | 89.45% | 87.91% |
| Radiation Therapy | 50% | 81.66% | 72.94% |
| Pancreatic Surgery | 86.49% | 85.55% | 74.53% |
| Respiratory and Critical Care Medicine | 100% | 78.05% | 70.09% |
| Colorectal Surgery | 80.65% | 58.60% | 58.22% |
| Gastrointestinal/Hernia Surgery | 84.89% | 82.81% | 73.75% |
| Ophthalmology | 94.34% | 85.25% | 93.30% |
| Gastrointestinal Oncology Surgery | 78.50% | 85.14% | 68.13% |
| Sports Medicine and Joint Surgery | 33.73% | 33.86% | 25.52% |
| Organ Transplantation and Hepatobiliary Surgery | 100% | 79.52% | 80.84% |
| Geriatric Endocrinology | 87.50% | 92.90% | 95% |
| Public Affairs | —— | 93.65% | 97.14% |
| Geriatric Cardiovascular Medicine | 60% | 76.79% | 75.46% |
| Hepatobiliary and Pancreatic Surgery | —— | 100% | 64.86% |
| Dermatology | 69.20% | 81.77% | 68.72% |
| Geriatric Neurology | —— | 89.66% | 91.35% |
| Infection Science | —— | 67.33% | 50.45% |
| Nephrology Peritoneal Dialysis | —— | 70.57% | 88.68% |
| Health Examination Center | 100% | 65.38% | 72.88% |
| International Physical Examination Center of Hunnan District | 82.35% | 72.73% | 52.05% |
| Clinical Pharmacology | —— | 88.70% | 55.74% |
| Pathology Diagnostics | —— | 78.31% | 62.75% |
| Rehabilitation Medicine I | —— | 87.43% | 70.45% |
| Anesthesiology | —— | 50% | 57.89% |
| Rehabilitation Medicine II | —— | 90% | 83.33% |
| Geriatric Gastroenterology | —— | 89.13% | 83.33% |
| Geriatric Comprehensive | 100% | 86.21% | 42.86% |

**Table S4.** Doctor-Patient Ratios by Departments

| Sub-discipline | 2021 | 2022 | 2023 |
| --- | --- | --- | --- |
| Traditional Chinese Medicine | 1：4 | 1：295 | 1：1654 |
| Vascular and Thyroid Surgery | 1：17 | 1：303 | 1：931 |
| Breast Surgery I | 1：93 | 1：543 | 1：890 |
| Psychology | —— | 1：508 | 1：867 |
| Endocrinology | 1：236 | 1：784 | 1：832 |
| Gastroenterology | 1：940 | 1：834 | 1：787 |
| Rheumatology and Immunology | 1：460 | 1：794 | 1：747 |
| Infectious Diseases | 1：358 | 1：687 | 1：710 |
| Breast Surgery III | 1：63 | 1：1107 | 1：610 |
| Cardiovascular Medicine | 1：135 | 1：630 | 1：587 |
| Thoracic Surgery | 1：153 | 1：306 | 1：556 |
| Thyroid Surgery | 1：72 | 1：351 | 1：536 |
| Hematology | 1：18 | 1：543 | 1：458 |
| Gynecology | 1：423 | 1：437 | 1：399 |
| Oncology II | —— | 1：1198 | 1：361 |
| Care Outpatient | 1：46 | 1：1219 | 1：353 |
| Nuclear Medicine | 1：153 | 1：289 | 1：345 |
| Plastic Surgery | 1：123 | 1：128 | 1：338 |
| Breast Surgery II | 1：120 | 1：539 | 1：333 |
| Nephrology | 1：28 | 1：258 | 1：311 |
| Pediatrics | 1：8 | 1：201 | 1：286 |
| Hepatobiliary Surgery | 1：310 | 1：202 | 1：283 |
| Orthopedics | 1：91 | 1：336 | 1：278 |
| Geriatric Respiratory and Infectious Diseases | 1：25 | 1：194 | 1：272 |
| Urology | 1：173 | 1：268 | 1：267 |
| Public Affairs | —— | 1：120 | 1：242 |
| Otorhinolaryngology | 1：58 | 1：138 | 1：221 |
| Hepatobiliary and Pancreatic Surgery | —— | 1：1 | 1：188 |
| Oncology | 1：11 | 1：1106 | 1：185 |
| Gastrointestinal/Hernia Surgery | 1：99 | 1：81 | 1：182 |
| Ultrasound Diagnostics | 1：17 | 1：132 | 1：174 |
| Sports Medicine and Joint Surgery | 1：235 | 1：375 | 1：165 |
| Neurology | 1：125 | 1：226 | 1：149 |
| Organ Transplantation and Hepatobiliary Surgery | 1：9 | 1：119 | 1：138 |
| Obstetrics | 1：79 | 1：232 | 1：125 |
| General Medicine | 1：50 | 1：114 | 1：119 |
| Cardiac Surgery | 1：28 | 1：64 | 1：118 |
| Interventional Medicine | 1：11 | 1：118 | 1：107 |
| Geriatric Cardiovascular Medicine | 1：10 | 1：55 | 1：105 |
| Pain Medicine | 1：47 | 1：123 | 1：82 |
| Clinical Pharmacology | —— | 1：657 | 1：76 |
| Nephrology Peritoneal Dialysis | —— | 1：396 | 1：74 |
| Neurosurgery | 1：42 | 1：82 | 1：72 |
| Proctology | 1：19 | 1：89 | 1：61 |
| Radiation Therapy | 1：1 | 1：124 | 1：53 |
| Geriatric Endocrinology | 1：18 | 1：65 | 1：51 |
| Pancreatic Surgery | 1：62 | 1：77 | 1：50 |
| Ophthalmology | 1：34 | 1：79 | 1：50 |
| Gastrointestinal Oncology Surgery | 1：25 | 1：129 | 1：47 |
| Geriatric Neurology | —— | 1：29 | 1：32 |
| Respiratory and Critical Care Medicine | 1：16 | 1：144 | 1：25 |
| Rehabilitation Medicine I | —— | 1：50 | 1：18 |
| Pathology Diagnostics | —— | 1：25 | 1：16 |
| International Physical Examination Center of Hunnan District | 1：22 | 1：72 | 1：13 |
| Infection Science | 1：12 | 1：23 | 1：11 |
| Dermatology | 1：32 | 1：19 | 1：10 |
| Anesthesiology | —— | 1：7 | 1：7 |
| Geriatric Gastroenterology | —— | 1：51 | 1：7 |
| Health Examination Center | 1：25 | 1：8 | 1：6 |
| Rehabilitation Medicine II | —— | 1：20 | 1：2 |
| Geriatric Comprehensive | 1：13 | 1：47 | 1：2 |
| Endoscopy | —— | 1：107 | —— |
| Geriatric | 1：1 | 1：29 | —— |
| Pharmacy Services | —— | 1：1 | —— |

**Table S5.** Consultation Volume Across Departments

| Rank | Department | 2021 | 2022 | 2023 |
| --- | --- | --- | --- | --- |
| 1 | Breast Surgery | 2220 | 18007 | 23741 |
| 2 | Gastroenterology | 7518 | 16673 | 14959 |
| 3 | Rheumatology and Immunology | 5062 | 14286 | 13441 |
| 4 | Oncology | 114 | 26909 | 4953 |
| 5 | Endocrinology | 1417 | 11763 | 14147 |
| 6 | Cardiovascular Medicine | 807 | 13854 | 11156 |
| 7 | Gynecology | 5071 | 7427 | 6788 |
| 8 | Thoracic Surgery | 1117 | 5818 | 11676 |
| 9 | Urology | 2593 | 6428 | 7740 |
| 10 | Neurology | 627 | 8810 | 5367 |
| 11 | Infectious Diseases | 1790 | 6187 | 6386 |
| 12 | Hematology | 110 | 7602 | 5952 |
| 13 | Traditional Chinese Medicine | 21 | 1771 | 11580 |
| 14 | Psychology | 0 | 4060 | 6933 |
| 15 | General Surgery | 1022 | 5344 | 4331 |
| 16 | Nephrology | 251 | 4525 | 4510 |
| 17 | Thyroid Surgery | 288 | 2103 | 5891 |
| 18 | Vascular Surgery | 17 | 2423 | 5587 |
| 19 | Care Outpatient | 46 | 6097 | 1767 |
| 20 | Plastic Surgery | 491 | 1151 | 4052 |
| 21 | Otorhinolaryngology | 288 | 2888 | 2426 |
| 22 | Pediatrics | 33 | 2206 | 3143 |
| 23 | Obstetrics | 551 | 2091 | 1620 |
| 24 | Nuclear Medicine | 765 | 1443 | 1724 |
| 25 | Orthopedics | 598 | 2053 | 1164 |
| 26 | Neurosurgery | 339 | 2060 | 1362 |
| 27 | Geriatrics | 148 | 1082 | 1662 |
| 28 | General Practice | 353 | 1026 | 1067 |
| 29 | Pain Medicine | 187 | 1229 | 899 |
| 30 | Ultrasonography | 51 | 661 | 1219 |
| 31 | Respiratory and Critical Care Medicine | 31 | 1443 | 402 |
| 32 | Radiation Therapy | 1 | 1367 | 475 |
| 33 | Ophthalmology | 238 | 1178 | 397 |
| 34 | Interventional Medicine | 11 | 944 | 534 |
| 35 | Cardiac Surgery | 56 | 575 | 825 |
| 36 | Clinical Pharmacology | 0 | 1314 | 76 |
| 37 | Surgical Oncology | 148 | 903 | 332 |
| 38 | Dermatology | 222 | 322 | 146 |
| 39 | Health Examination Center | 91 | 300 | 95 |
| 40 | Rehabilitation Medicine | 0 | 349 | 45 |
| 41 | Endoscopy | 0 | 214 | 0 |
| 42 | Infection | 23 | 113 | 56 |
| 43 | Pathology | 0 | 124 | 31 |
| 44 | Anesthesiology | 0 | 20 | 21 |

**Table S6.** Online vs. Offline Outpatient Appointment Ratios by Department

| Department | Year | Outpatient Appointments | Proportion of Online Appointments | Proportion of Offline Appointments |
| --- | --- | --- | --- | --- |
| Pathology Diagnostics | 2021 | 16456 | 15.99% | 83.19% |
|  | 2022 | 15894 | 92.45% | 7.46% |
|  | 2023 | 17860 | 99.57% | 0.43% |
| Ultrasound Diagnostics | 2021 | 16378 | 0.12% | 99.88% |
|  | 2022 | 12664 | 72.94% | 27.06% |
|  | 2023 | 17585 | 99.81% | 0.19% |
| Care Outpatient | 2021 | 30777 | 46.14% | 53.46% |
|  | 2022 | 25532 | 66.36% | 33.50% |
|  | 2023 | 31035 | 66.64% | 33.36% |
| Nuclear Medicine | 2021 | 12109 | 82.52% | 16.56% |
|  | 2022 | 7478 | 98.45% | 1.14% |
|  | 2023 | 10523 | 99.37% | 0.60% |
| Thyroid Surgery | 2021 | 65181 | 81.38% | 15.87% |
|  | 2022 | 35609 | 97.01% | 2.61% |
|  | 2023 | 55602 | 99.67% | 0.33% |
| Health Management | 2021 | 52541 | 0.00% | 100.00% |
|  | 2022 | 33250 | 0.00% | 100.00% |
|  | 2023 | 47677 | 0.00% | 100.00% |
| Anesthesiology | 2021 | 16475 | 37.37% | 62.34% |
|  | 2022 | 14330 | 92.44% | 7.48% |
|  | 2023 | 21418 | 95.85% | 4.11% |
| Obstetrics | 2021 | 18270 | 57.30% | 41.22% |
|  | 2022 | 17844 | 91.62% | 8.13% |
|  | 2023 | 12543 | 98.79% | 1.20% |
| Pediatrics | 2021 | 32695 | 31.46% | 66.06% |
|  | 2022 | 21166 | 86.67% | 12.89% |
|  | 2023 | 41467 | 99.81% | 0.14% |
| Otorhinolaryngology | 2021 | 121055 | 68.25% | 31.12% |
|  | 2022 | 118759 | 97.15% | 2.77% |
|  | 2023 | 155620 | 99.61% | 0.29% |
| Radiation Therapy | 2021 | 14370 | 41.02% | 58.37% |
|  | 2022 | 15716 | 91.22% | 8.74% |
|  | 2023 | 12802 | 91.69% | 8.30% |
| Rheumatology and Immunology | 2021 | 55984 | 81.79% | 16.04% |
|  | 2022 | 33033 | 95.40% | 4.05% |
|  | 2023 | 60513 | 97.62% | 2.30% |
| Gynecology | 2021 | 109765 | 87.60% | 8.95% |
|  | 2022 | 86858 | 97.93% | 1.51% |
|  | 2023 | 109297 | 99.66% | 0.29% |
| Hepatobiliary and Pancreatic Surgery | 2021 | 7541 | 84.74% | 14.87% |
|  | 2022 | 6224 | 98.91% | 1.06% |
|  | 2023 | 8596 | 99.81% | 0.10% |
| Infectious Diseases | 2021 | 6053 | 64.53% | 33.92% |
|  | 2022 | 8773 | 97.24% | 2.36% |
|  | 2023 | 10123 | 99.63% | 0.35% |
| Colorectal Surgery | 2021 | 23139 | 73.11% | 25.52% |
|  | 2022 | 17947 | 97.67% | 1.61% |
|  | 2023 | 23127 | 99.71% | 0.19% |
| Orthopedics | 2021 | 84567 | 76.50% | 19.95% |
|  | 2022 | 54781 | 96.36% | 2.84% |
|  | 2023 | 76185 | 99.64% | 0.20% |
| Respiratory and Critical Care Medicine | 2021 | 52766 | 81.37% | 17.53% |
|  | 2022 | 38258 | 96.70% | 3.03% |
|  | 2023 | 34628 | 99.60% | 0.31% |
| Interventional Medicine | 2021 | 9662 | 71.44% | 27.08% |
|  | 2022 | 10705 | 98.13% | 1.62% |
|  | 2023 | 11466 | 99.63% | 0.35% |
| Rehabilitation Medicine II | 2021 | 4337 | 24.12% | 75.35% |
|  | 2022 | 4932 | 84.45% | 15.41% |
|  | 2023 | 4293 | 92.83% | 7.15% |
| Rehabilitation Medicine I | 2021 | 4482 | 28.16% | 71.35% |
|  | 2022 | 6054 | 89.76% | 10.24% |
|  | 2023 | 5806 | 92.68% | 7.30% |
| Geriatric Comprehensive | 2021 | 469 | 69.08% | 30.49% |
|  | 2022 | 879 | 97.16% | 2.84% |
|  | 2023 | 2173 | 99.26% | 0.64% |
| Urology | 2021 | 84527 | 58.23% | 38.91% |
|  | 2022 | 69357 | 95.63% | 3.81% |
|  | 2023 | 88385 | 98.97% | 0.95% |
| Endocrinology | 2021 | 100942 | 67.30% | 30.50% |
|  | 2022 | 65097 | 96.06% | 3.55% |
|  | 2023 | 108173 | 94.54% | 5.38% |
| Endoscopy | 2021 | 20669 | 10.72% | 89.27% |
|  | 2022 | 17191 | 14.43% | 85.57% |
|  | 2023 | 21224 | 24.14% | 75.86% |
| Dermatology | 2021 | 151123 | 79.68% | 17.04% |
|  | 2022 | 122764 | 96.88% | 2.45% |
|  | 2023 | 186875 | 98.38% | 1.53% |
| Hepatobiliary Surgery | 2021 | 22948 | 82.20% | 15.60% |
|  | 2022 | 16712 | 96.85% | 2.35% |
|  | 2023 | 23392 | 99.71% | 0.20% |
| Organ Transplantation and Hepatobiliary Surgery | 2021 | 14481 | 43.42% | 54.98% |
|  | 2022 | 13424 | 89.68% | 10.04% |
|  | 2023 | 16828 | 98.45% | 1.55% |
| Breast Surgery II | 2021 | 15477 | 59.39% | 38.42% |
|  | 2022 | 9439 | 93.38% | 6.09% |
|  | 2023 | 19699 | 92.30% | 7.59% |
| Breast Surgery I | 2021 | 52127 | 64.31% | 34.22% |
|  | 2022 | 31033 | 94.88% | 4.77% |
|  | 2023 | 45570 | 96.83% | 3.12% |
| Breast Surgery III | 2021 | 20729 | 69.43% | 24.63% |
|  | 2022 | 20799 | 96.30% | 2.96% |
|  | 2023 | 37184 | 95.99% | 4.01% |
| Neurology | 2021 | 88270 | 77.92% | 18.86% |
|  | 2022 | 59617 | 94.31% | 4.90% |
|  | 2023 | 87067 | 99.34% | 0.50% |
| Neurosurgery | 2021 | 21056 | 81.89% | 14.98% |
|  | 2022 | 24854 | 98.23% | 1.29% |
|  | 2023 | 26280 | 99.41% | 0.50% |
| Nephrology | 2021 | 74270 | 84.38% | 14.17% |
|  | 2022 | 47268 | 97.89% | 1.79% |
|  | 2023 | 58471 | 99.69% | 0.25% |
| Gastrointestinal Surgery | 2021 | 13326 | 81.29% | 15.74% |
|  | 2022 | 8811 | 97.06% | 2.30% |
|  | 2023 | 13136 | 99.57% | 0.27% |
| Gastroenterology | 2021 | 103201 | 85.92% | 10.54% |
|  | 2022 | 63877 | 96.70% | 2.57% |
|  | 2023 | 108330 | 99.67% | 0.21% |
| Cardiovascular Medicine | 2021 | 106354 | 59.44% | 37.54% |
|  | 2022 | 69065 | 94.45% | 4.78% |
|  | 2023 | 88975 | 96.99% | 2.93% |
| Cardiac Surgery | 2021 | 10676 | 69.45% | 27.82% |
|  | 2022 | 7851 | 98.24% | 1.10% |
|  | 2023 | 9537 | 99.58% | 0.35% |
| Thoracic Surgery | 2021 | 44547 | 66.04% | 31.82% |
|  | 2022 | 36553 | 96.30% | 3.20% |
|  | 2023 | 49984 | 99.71% | 0.19% |
| Vascular and Thyroid Surgery | 2021 | 46164 | 87.67% | 10.84% |
|  | 2022 | 27755 | 97.02% | 2.42% |
|  | 2023 | 42862 | 99.63% | 0.28% |
| Hematology | 2021 | 38180 | 61.82% | 37.36% |
|  | 2022 | 26070 | 91.48% | 8.30% |
|  | 2023 | 35140 | 98.73% | 1.17% |
| Ophthalmology | 2021 | 72373 | 61.09% | 37.65% |
|  | 2022 | 60641 | 96.99% | 2.74% |
|  | 2023 | 86782 | 99.39% | 0.51% |
| Hepatobiliary Surgery | 2021 | 10620 | 65.34% | 33.31% |
|  | 2022 | 10395 | 98.57% | 1.03% |
|  | 2023 | 11635 | 99.72% | 0.21% |
| Sports Medicine and Joint Surgery | 2021 | 25595 | 71.46% | 26.02% |
|  | 2022 | 22620 | 97.59% | 1.95% |
|  | 2023 | 28209 | 99.51% | 0.41% |
| Plastic Surgery | 2021 | 7512 | 52.56% | 46.38% |
|  | 2022 | 8579 | 97.61% | 2.14% |
|  | 2023 | 12424 | 99.69% | 0.21% |
| Traditional Chinese Medicine | 2021 | 67259 | 24.26% | 75.10% |
|  | 2022 | 60294 | 97.11% | 2.61% |
|  | 2023 | 55889 | 97.45% | 2.50% |
| Oncology | 2021 | 41509 | 70.06% | 27.97% |
|  | 2022 | 23689 | 91.20% | 8.40% |
|  | 2023 | 29404 | 94.22% | 5.70% |
| Oncology II | 2021 | 23425 | 94.16% | 5.38% |
|  | 2022 | 16697 | 97.31% | 2.61% |
|  | 2023 | 18997 | 97.85% | 2.13% |
| General Medicine | 2021 | 11769 | 49.91% | 47.30% |
|  | 2022 | 9704 | 97.54% | 1.99% |
|  | 2023 | 14109 | 99.45% | 0.46% |
| Nephrology Peritoneal Dialysis | 2021 | 2058 | 0.92% | 99.08% |
|  | 2022 | 1616 | 67.08% | 32.92% |
|  | 2023 | 2306 | 97.88% | 2.12% |
| Pain Management | 2021 | 17292 | 55.26% | 42.35% |
|  | 2022 | 14376 | 92.36% | 7.13% |
|  | 2023 | 18736 | 99.35% | 0.56% |
| Gastrointestinal/Hernia Surgery | 2021 | 15354 | 64.13% | 33.87% |
|  | 2022 | 12197 | 98.45% | 1.22% |
|  | 2023 | 13078 | 99.83% | 0.03% |
| Psychology | 2021 | 52268 | 90.65% | 6.94% |
|  | 2022 | 32078 | 96.35% | 2.97% |
|  | 2023 | 41921 | 98.89% | 1.02% |
| Geriatric Respiratory and Infectious Diseases | 2021 | 599 | 58.76% | 41.07% |
|  | 2022 | 394 | 93.40% | 6.35% |
|  | 2023 | 1006 | 99.70% | 0.20% |
| Geriatric Neurology | 2021 | 876 | 77.17% | 21.69% |
|  | 2022 | 1108 | 97.92% | 1.90% |
|  | 2023 | 2723 | 99.71% | 0.22% |
| Geriatric Gastroenterology | 2021 | 78 | 44.87% | 55.13% |
|  | 2022 | 532 | 97.56% | 2.44% |
|  | 2023 | 564 | 98.05% | 1.95% |
| Geriatric Cardiovascular Medicine | 2021 | 1165 | 58.63% | 41.20% |
|  | 2022 | 1022 | 98.53% | 1.47% |
|  | 2023 | 2384 | 99.83% | 0.13% |
| International Physical Examination Center of Hunnan District | 2021 | 350 | 0.00% | 100.00% |
|  | 2022 | 832 | 0.00% | 100.00% |
|  | 2023 | 1162 | 0.00% | 100.00% |
| Health Examination Center | 2021 | 206 | 26.70% | 73.30% |
|  | 2022 | 183 | 100.00% | 0.00% |
|  | 2023 | 262 | 100.00% | 0.00% |
| Geriatric | 2021 | 3 | 0.00% | 100.00% |
|  | 2022 | 1 | 100.00% | 0.00% |
|  | 2023 | 0 | —— | —— |
| Geriatric Endocrinology | 2021 | 362 | 62.15% | 36.46% |
|  | 2022 | 1056 | 97.54% | 2.46% |
|  | 2023 | 1480 | 99.32% | 0.68% |
| Clinical Pharmacology | 2021 | 281 | 0.00% | 100.00% |
|  | 2022 | 2436 | 84.56% | 15.44% |
|  | 2023 | 4858 | 99.98% | 0.02% |
| Health Management | 2021 | 0 | —— | —— |
|  | 2022 | 2117 | 91.88% | 8.12% |
|  | 2023 | 1351 | 84.53% | 15.47% |
| Infectious Diseases | 2021 | 0 | —— | —— |
|  | 2022 | 0 | —— | —— |
|  | 2023 | 59 | 98.31% | 1.69% |

**Table S7.** Descriptions of Service Function for SHIIP

| Function | Description |
| --- | --- |
| Health checkup appointment | To provide physical examination appointment services for healthy individuals on mobile and other mobile devices. |
| Health self-assessment and screening | To provide self-health and disease risk assessment services by using assessment forms and other methods. |
| Online payment | To enable payment for physical examination services via mobile and other mobile devices. |
| View results | To view the results of laboratory tests and other examinations conducted during a physical examination. |
| Checkup report analysis | To offer online interpretation and guidance services for physical examination results for examinees |
| Health education | To provide online health education and promotion for the general population . |
| Smart healthcare navigation | To provide answers to questions about medical procedures, clinical navigation, and related topics with intelligent chatbots. |
| Intelligent pre-diagnosis triage | To collect pre-diagnosis information and provide automatic triage recommendations by using intelligent chatbots . |
| Appointment registration | To make appointments for offline clinic appointments through online platforms. |
| Online payment | To pay for registration, medical advice, and other related expenses through mobile devices such as smartphones. |
| Examination and test appointment | To provide online appointment booking services for medical procedures such as examinations, tests, and treatments. |
| View results | To enable patients to view their examination, testing, diagnosis, treatment advice, and other medical information online. |
| Emergency triage and pre-diagnosis | To provide online pre-diagnosis and triage services for emergency cases, to reduce the time needed for preliminary treatment. |
| Emergency response collaboration | To enable online coordination among all stages of the emergency green channel process. |
| Collaborative critical patient transport | Realizing collaborative services such as remote mobile consultations during the process of severe patient transfer. |
| PCP enrollment services | Collaborating with community doctors to provide online health management services for signed family doctors. |
| Remote consultation | To provide online video consultation services for patients in lower-level hospitals. |
| Bidirectional referral | To collaborate with lower-level hospitals to complete online two-way referrals. |
| Regular follow-up management | To perform automated follow-up plans and progress monitoring through intelligent voice calls and forms. |
| Condition monitoring | Checking and tracking patients’ health status online over time to identify any changes or issues that may require intervention. |
| Medication management | To online ensure safe, effective, and appropriate use of medications by patients. |
| Event reminder and alert | To provide patients with automatic and intelligent reminders for medication management and follow-up appointments. |
| Disease education | To educate patients online about their condition, treatment options, and how to manage their disease effectively. |
| Lifestyle management | Online Guiding and helping patients to make healthy lifestyle choices, such as a healthy diet and regular exercise. |
| Online medical consultation | To provide medical advice, diagnosis, and treatment recommendations to online patients. |
| Online payment | To pay for online Internet hospital visit and medical advice-related expenses through patients' mobile devices. |
| Examination and test appointment | Realizing the online appointment of examinations, laboratory tests, treatments and other services in internet hospitals. |
| View results | To view diagnostic, treatment recommendations, laboratory and imaging test results in internet hospitals. |
| Medication postal delivery | Providing online prescription and medication delivery service for patients of internet hospitals. |
| Hospital admission booking | To provide online hospitalization appointment service for patients of internet hospitals. |
| Hospital navigation | To facilitate wayfinding and guidance within the hospital premises for patients and visitors. |
| Online ordering | To facilitate convenient food ordering through an online platform for patients' convenience |
| Online shopping mall | To provide convenient online purchasing of medical products and services within the Internet hospital. |
| Parking payment | To facilitate convenient parking payment for visitors in Internet hospitals. |
| Companion appointment | To schedule a companion to accompany during medical visits in Internet hospitals. |
| Electronic invoice | To streamline billing and record-keeping, Internet hospitals offer Electronic invoicing. |

**Table S8.** Descriptions of Medical Function

| Function | Description |
| --- | --- |
| Visit management | Healthcare professionals can independently manage their online hospital visit schedule, including the time, quantity, and whether it is free. |
| Antibiotic review | Senior physicians with antibiotic review qualifications can complete offline antibiotic review work on their mobile APP. |
| Consultation management | Physicians responsible for consultations can view and process pending offline consultation cases on their mobile APP. |
| Publication of medical education | Healthcare professionals can edit, save, and publish medical education articles for patient education on their mobile devices. |
| Offline clinic appointment | Outpatient physicians can use the mobile APP to add appointments for patients when the offline clinic schedule is fully booked. |
| Offline system login | Healthcare professionals can directly log into the offline HIS using the mobile APP by scanning a QR code. |
| Unified management | Enabling physicians to have unified management of their outpatient, inpatient, and online hospital patients. |
| Patient grouping | Healthcare professionals can group patients on their mobile APP for easier follow-up, monitoring, and communication. |
| Patient labeling | Healthcare professionals can create custom labels on their mobile APP and apply them to patients for easy identification and categorization. |
| Message sending | Healthcare professionals can send various messages to patients individually or in groupings, such as follow-up reminders or clinic closure notices. |
| Research follow-up | To enable automated follow-up based on patient characteristics and custom rules, using electronic forms and automated phone calls, among other methods. |
| Patient recruitment | To facilitate patient recruitment, information collection, and group management for clinical research projects. |
| Historical medical records | Healthcare professionals can view a patient's historical medical information, including outpatient and inpatient records, on their mobile APP. |
| Prescription information | Doctors can check the status of prescriptions they issued in the online hospital, such as pending pharmacist review or already reviewed. |
| Test and laboratory results | Healthcare professionals can access a patient's laboratory and test results through their mobile APP. |
| Surgery progress information | Surgeons can view the progress of surgeries in their specialty and estimate the time for their own surgeries, facilitating preparation. |
| Critical value alerts | The system can provide doctors with alerts and access to critical values for the patients they manage. |
| Colleague sharing | The system supports online communication among healthcare professionals, allowing them to view colleagues' scientific knowledge sharing and other content. |
| Pre-consultation data collection | The platform utilizes pre-consultation data collected from patients to generate electronic medical records, reducing the burden on doctors. |
| Refusal of consultation | The platform optimizes the workflow and provides doctors with a one-click option to refuse a consultation, cancel an appointment, and issue refunds. |
| Prescription recommendation | The system utilizes CDSS to provide prescription recommendations for doctors in the online consultation setting. |
| Quick replies | The platform configures customized quick reply options within the consultation dialogue box to improve work efficiency for doctors. |
| Privacy phone calls | To enable direct access to privacy phone calls, facilitating voice communication between doctors and patients when text-based communication is insufficient. |
| Medical guidance | To deliver various medical knowledge and guidance to patients prior to consultations, improving communication efficiency between doctors and patients. |

**Table S9.** Descriptions of Management Support Function

| Function | Description |
| --- | --- |
| Qualification application | Healthcare professionals can submit their online applications for attending Internet hospital visits, filling in and uploading relevant information. |
| Qualification review | Medical management personnel can review online whether the applicants possess the qualifications for Internet hospital visits. |
| Visit training | To train for prospective visiting doctors on legal, professional, and platform usage aspects by a combination of online and offline methods. |
| Qualification assessment | Online assessment of doctors' qualifications related to Internet visits is conducted through a question-and-answer format before granting authorization for visits. |
| Visit registration | Qualifications and related information of visiting personnel are automatically transmitted through data integration with regulatory platforms. |
| Information modification | Healthcare professionals can apply, approve, and modify their personal information online using the platform. |
| Data monitoring | The platform visually presents relevant operational data of the Internet hospital through visualized data display. |
| Data analysis | Utilizing the platform to perform statistical analysis on various data to guide adjustments in construction and operational strategies. |
| Decision support | AI models for management decision support using operational data of the Internet hospital to explore the establishment of an Internet hospital brain. |
| Data Reporting | Real-time transmission of Internet-based medical treatment data to provincial regulatory platforms through data integration. |
| Medical quality control | Digitally monitoring the quality of physician diagnoses, prescription issuance, medical record writing, and service quality on the platform. |
| Data publication | Regularly publishing statistics on departmental and individual patient visits to stimulate performance comparisons. |
| Load balancing monitoring | It involves ensuring the proper distribution of requests and traffic across backend servers, optimizing system performance. |
| System performance monitoring | It involves monitoring critical metrics such as system's operational status, resource utilization, and response time. |
| Log and event monitoring | It involves monitoring system logs and event information in real-time to identify and resolve potential issues. |
| Disaster recovery and backup | It involves implementing a robust backup and recovery system to mitigate risks of system failures, disasters, and data loss. |
| Data interface monitoring | It involves monitoring the data exchange processes between different systems or applications to ensure stable data transmission and accuracy. |
| Information security monitoring | It involves monitoring the security and confidentiality of the system, as well as detecting and preventing potential security threats. |

**Figure S1. The architecture of overall framework of SHIIP.**


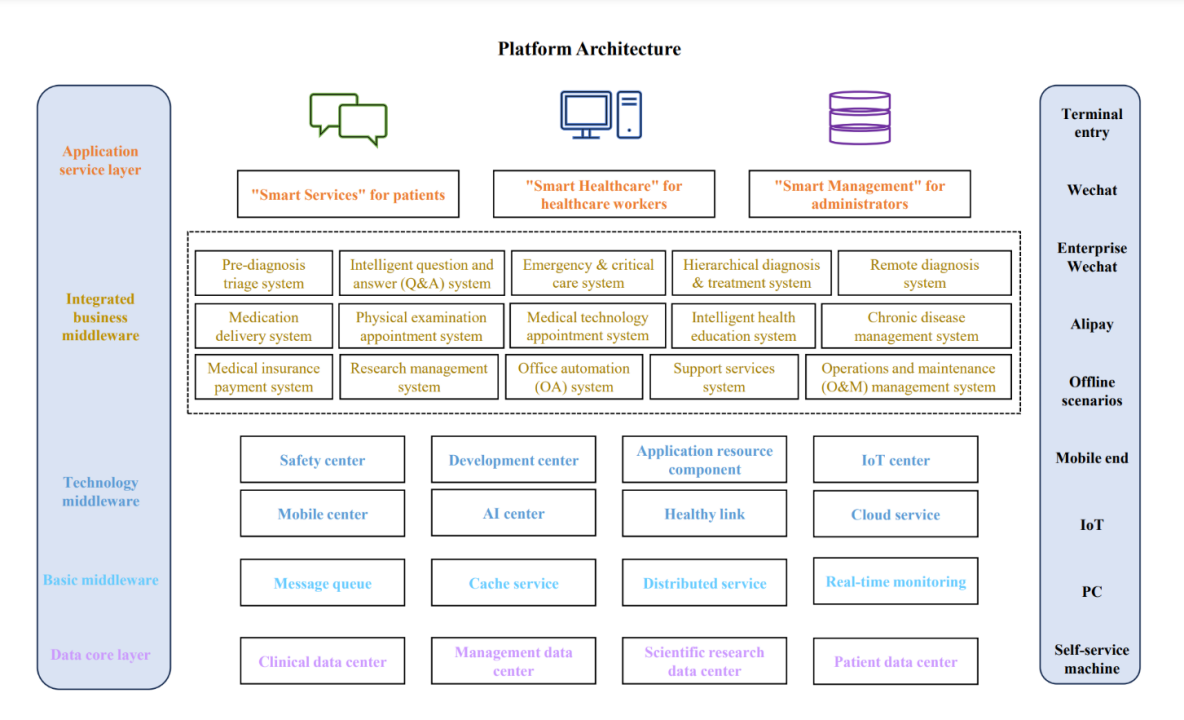


This architecture is elaborated in five technical levels with different colors respectively. SHIIP: Smart Hospital Intelligent Internet Platform; AI: Artificial Intelligence; LoT: Internet of Things; PC: Personal computer.

**Figure S2. Technical Architecture of the SHIIP**


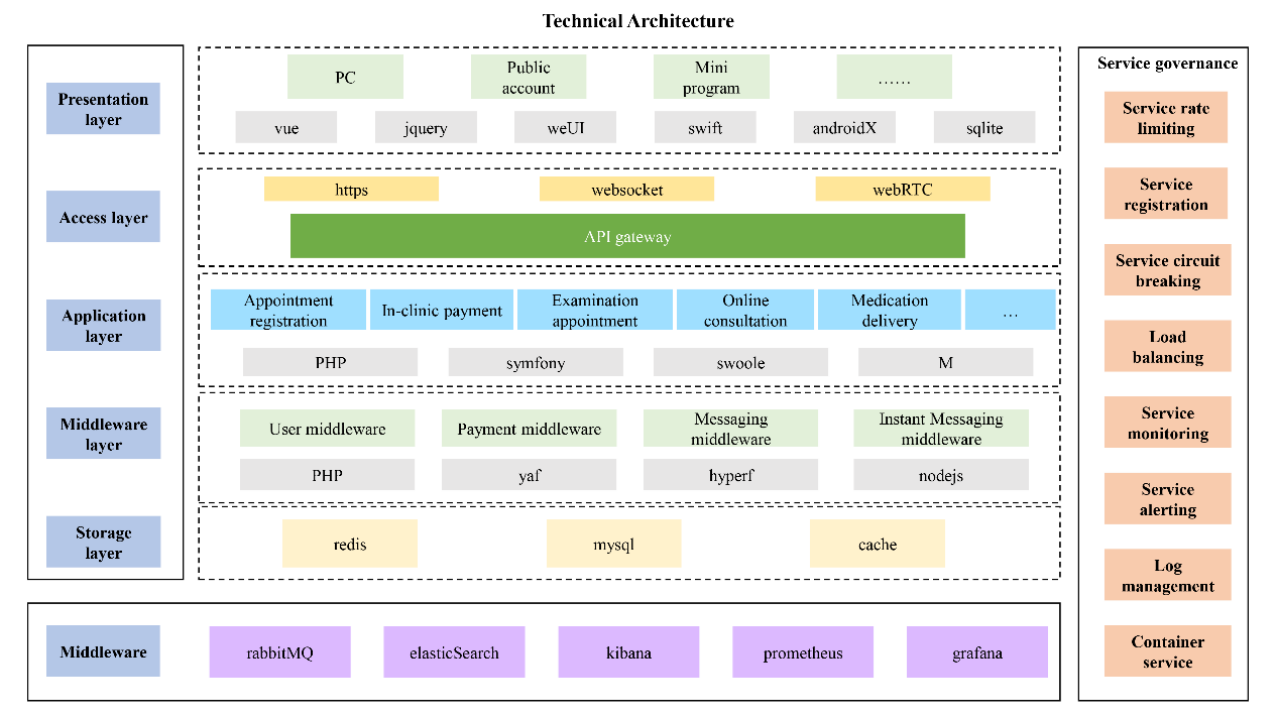


The figure illustrates the technical architecture of the SHIIP. Abbreviation: PC, Personal Computer; Vue, a JavaScript framework; jQuery, a JavaScript library; WeUI, a UI library; Swift, a programming language; AndroidX, a package of Android libraries; SQLite, a lightweight and embedded database management system; API, Application Programming Interface; PHP, Hypertext Preprocessor; Symfony, a PHP web application framework; Swoole, a high-performance PHP coroutine framework; M, a system development language; Yaf, a PHP framework; Hyperf, a high-performance PHP coroutine framework; Node.js, a JavaScript runtime environment; Redis, an in-memory data structure store; MySQL, A relational database management system; Cache: a databases for caching data; RabbitMQ, a message broker that implements the Advanced Message Queuing Protocol (AMQP); Elasticsearch, a distributed, RESTful search and analytics engine; Kibana, an open-source data visualization and exploration tool for Elasticsearch; Prometheus, a monitoring and alerting toolkit; Grafana, an open-source analytics and monitoring solution for visualizing metrics.

**Figure S3. Network Topology Logical Structure Diagram of the SHIIP**


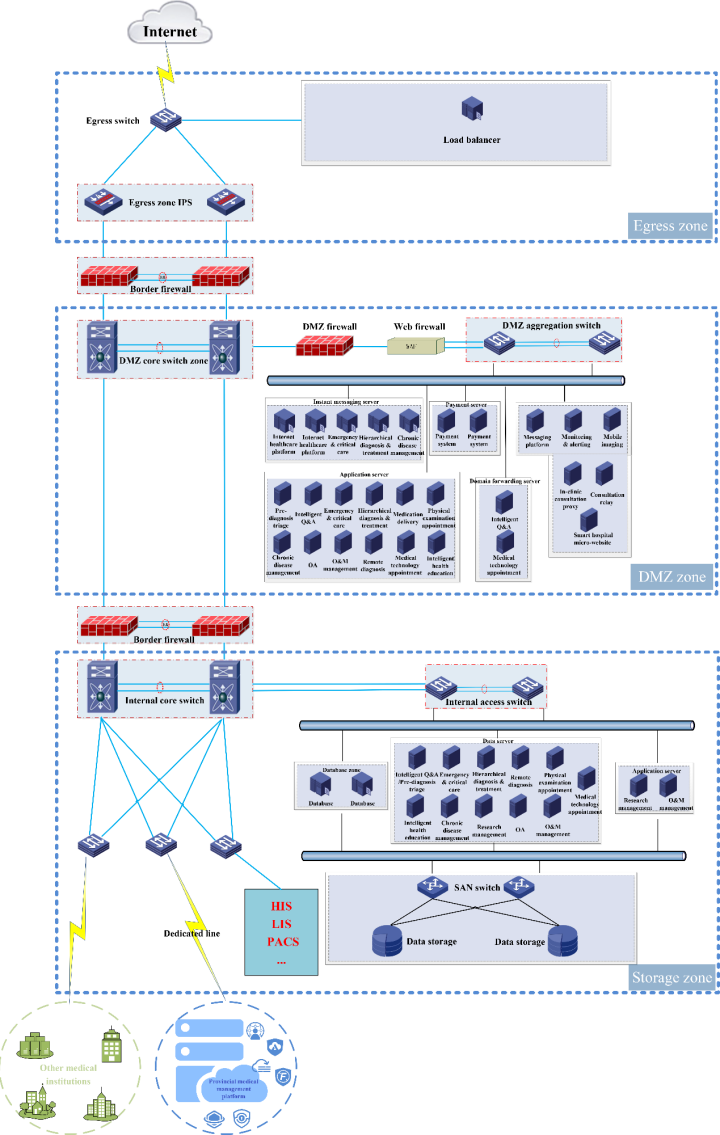


The figure depicts the logical structure of the network topology used in the construction of the SHIIP. Abbreviation: IPS, Intrusion Prevention System; DMZ, Demilitarized Zone; WAF, Web Application Firewall; HA, High Availability; HIS: Hospital Information System; LIS, Laboratory Information System; PACS, Picture Archiving and Communication System; SAN, Storage Area Network.

**Figure S4. Data Service Architecture of the SHIIP**


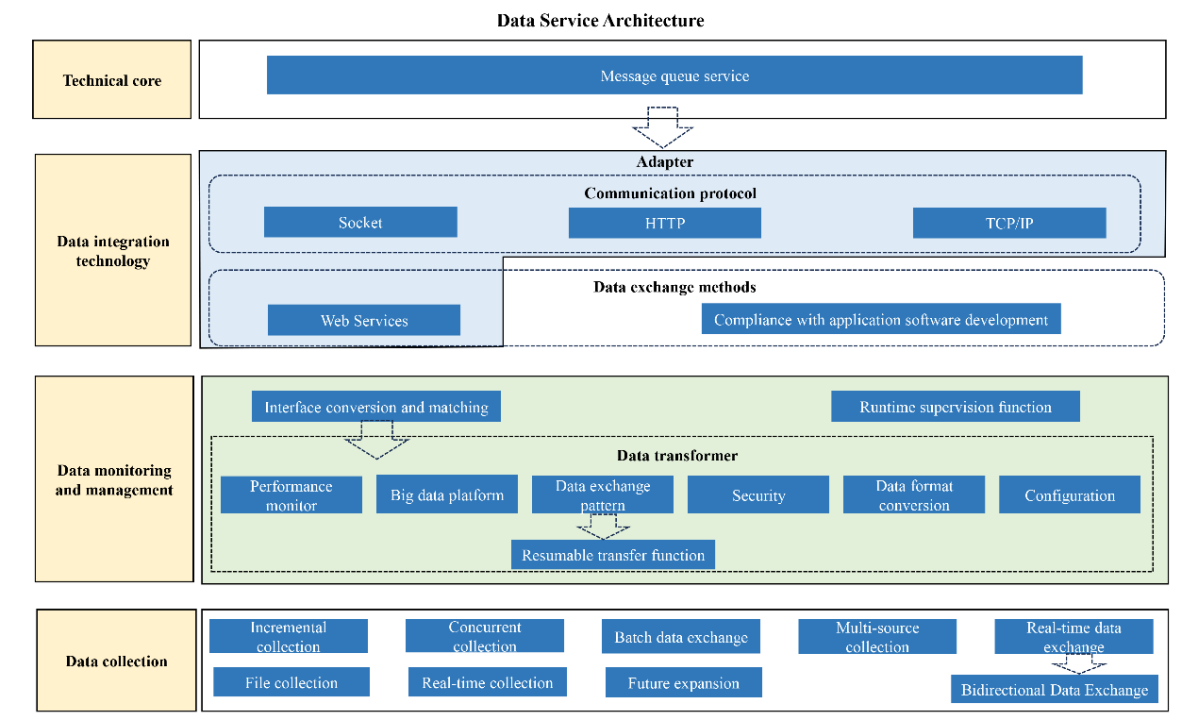


The figure illustrates the data service system architecture of the SHIIP. Abbreviations: HTTP, Hypertext Transfer Protocol; TCP/IP: Transmission Control Protocol/Internet Protocol.
